# Sex-specific Effects of Music Listening on Couples’ Stress in Everyday Life

**DOI:** 10.1038/s41598-019-40056-0

**Published:** 2019-03-19

**Authors:** A. Wuttke-Linnemann, U. M. Nater, U. Ehlert, B. Ditzen

**Affiliations:** 1 University Medical Center Mainz, Mainz, Germany; 2 University of Vienna, Vienna, Austria; 3 University of Zurich, Zurich, Switzerland; 4 University Hospital, Heidelberg University, Heidelberg, Germany; 5 Center for Mental Health in Old Age, Landeskrankenhaus (AöR), Mainz, Germany

## Abstract

Music listening in daily life is associated with stress-reducing effects on the individual with increasing effects when music listening occurs in a social context. As little is known about effects on couples, we investigated whether beneficial effects can be found in couples. Forty heterosexual couples were investigated using ambulatory assessment. Participants completed six assessments on music listening and subjective stress per day for five consecutive days. With each assessment, saliva samples for the later analysis of cortisol and alpha-amylase were collected. Music listening affected biopsychological stress markers in women and men, however in different ways: While music listening reduced cortisol in women, it increased alpha-amylase in men. Dyadic effects of music listening on stress markers were found. Men showed lower secretion of cortisol if women listened to music which was more pronounced when couples shared musical preferences. Both men and women showed higher alpha-amylase activity when their partner had listened to music. Music listening influences couples’ psychobiological stress levels in a sex-dependent manner with evidence of dyadic co-variation in physiological responses to music. Interventions for promoting stress reduction should consider that women and men differ in their use of music in everyday life.

## Introduction

Stress is a threat to health. Whereas acute stress is adaptive, chronic stress can lead to health deteriorations^1^. Mechanisms underlying these detrimental effects posit that through chronic activation of stress-sensitive systems in the body interacting with the immune system negative effects of stress on health can be explained^1^. In this regard, the two major stress-sensitive systems in the body - the hypothalamic–pituitary–adrenal (HPA) axis and the autonomic nervous system (ANS) - are of special interest. The HPA axis is responsible for the secretion of cortisol which is linked to the experience of stress. Higher levels of cortisol can suppress immune system functioning and this negatively affects health. Whereas peak levels of cortisol can be found roughly 20 minutes after a stressor, the ANS shows more immediate effects in reaction to stress. With onset of a stressor, activations of ANS lead to increases in heart rate, skin conductance and activity of the enzyme alpha-amylase. Alpha-amylase has been found to be an indicator for sympathetic nervous system activity related to health-relevant aspects as well^2,3^. Especially with stress being present in daily life, it is important to (a) find means to reduce stress in daily life and (b) to understand mechanisms underlying these effects. Music listening as a ubiquitous and popular activity in daily life might be one avenue to beneficially affect stress and promote health in daily life.

There is a long tradition in associating music listening with health benefits. It is proposed that these effects are mediated by a reduction in psychobiological stress^4^. Findings from experimental studies show that psychophysiological effects of music listening are linked to characteristics of the music, the situation, and the person^5^. In three recent reviews^4,6,7^, psychobiological mechanisms underlying the effects of music listening have been reviewed based on experimental studies with evidence pointing to music listening being associated with a down-regulation of HPA axis and ANS activity and beneficial effects on immune system activity. In an attempt to validate these findings in an ecological valid setting, research started investigating the effects of music listening in daily life. Indeed, music listening in daily life has shown stress-reducing effects^8,9^. However, it might not be music per se that exerts a stress-reducing effect. Rather, non-musical characteristics seem to mediate this relationship. Recently, it was shown that the social context of the listening situation has an important impact on this stress-reducing effect in daily life as music listening in the presence of others (as compared to music listening in solitude) was associated with attenuated stress levels^9^ and increases in positive affect^10^.

Social integration and close social relationships in general are associated with long-term health benefits^11^. Research on mechanisms underlying the health benefits of social relationships proposes that social support buffers the detrimental effects of stress on health^12^. This protective effect seems to be mediated by the activity of HPA axis and the ANS. Especially being in a stable romantic relationship has been previously linked to health benefits, as married couples show long-term health improvement in comparison to unmarried individuals^13^. In line with this, being married and high relationship quality in particular seem to have stress-reducing effects in daily life^14,15^. At the same time, stressful interactions between partners are thought to be particularly harmful. Dyadic data analysis suggests that in couples each partner’s (that in couples each partner’s) evaluations of marital quality (are also modulated by the) stress (of their partner) are also modulated by the affect of their partner, a phenomenon referred to as stress crossover^16^. Therefore, it can be assumed that couples can benefit from stress-reducing interventions which explicitly target the dyad as a unit. Positive couple interactions could be one stress-reducing strategy as these have been associated with reduced levels of cortisol and subjective stress on both members of the dyad^17^.

Music is associated with social interactions as well: It is assumed that engaging in music activities leads to social cohesion by facilitating contact, communication, coordination, and cooperation among others^18^. However, the beneficial effects of actively engaging in musical activities might not be limited to playing an instrument but also to music listening. Findings from neuroimaging studies support this notion as music listening shares neural networks that are critical to the perception and production of language^19^. Thus, music listening might be regarded as a nonverbal form of communication^20^. This attributes to music a promising role in the formation and maintenance of social relationships as music listening is considered an agent of social bonding and affiliation^21,22^. Whereas most research focused on the role of music listening for the formation of friendships and peer groups^23,24^, evidence on social functions of music listening in couples remains scarce and it is unclear whether results concerning friendships and peer groups can be readily transferred to couples.

So far, most research on the effects of music listening in a dyadic context focused on music therapy interventions either targeting couples of which one partner was hospitalized due to a medical condition or targeting couples seeking marriage counseling. Evidence from these studies suggests that music listening has positive effects on couple interaction^25^ providing a hint that music may act as an important facilitator for communication and connection in couples^20,25–27^. Furthermore, studies show that music listening in couples does not only improve communicative skills but also leads to relaxation^26,28^.

Consequently, there is a set of musical techniques available to help couples: These techniques vary from specialized music therapy programs for couples^20,27^ to individual music interventions, such as drumming, instrumental communication or body music^29^. However, these interventions are time limited and require a therapist^29^. Also, the question arises of how the effects might be translated to daily life experiences. Specifically, it would be relevant to know whether music listening, as opposed to music interventions, can have beneficial effects, too. An attempt to investigate the benefit from music in daily life was done by Hanser *et al*.^28^ who developed home-based music strategies for patients with dementia and their caregivers. A music therapist custom-tailored a music CD and participants received instructions to listen to the music and to talk about music-evoked memories. This intervention led to stress reduction in the caregivers. However, this study was just exploratory in nature, as only a small number of dyads were investigated. No data on the role of music preferences for the effects of music listening in couples are available, so far. However, this is particularly important, as from early childhood onwards, similar music preferences are associated with social bonding. For example, children seek the presence of other children with similar music preferences^30^ and same musical preferences are critical to social bonding in peers during adolescence^23^. However, at the same time, gender differences in musical preferences are consistently reported across studies^31,32^. Thus, it remains unknown what role similar music preferences play in couples and it is unknown if benefits of music listening in couples depend on the similarity of musical preferences.

Taken together, music therapy interventions can be an effective way to reduce stress in couples. However, music therapy interventions are time-intensive, require professional assistance, and have so far mostly been investigated in couples with a medical condition. Furthermore, most studies are qualitative or exploratory in nature and rely on self-report. Systematic research on the potential physiological mechanisms which might mediate stress-reducing effects of music in couples does not exist. Furthermore, there is only preliminary evidence that the effects of targeted therapeutic interventions might be generalized to daily life. There are two research questions which might follow: First, does music listening in daily life have beneficial effects in healthy couples? That is, does music listening in daily life reduce stress? Second, what role do (similar) music preferences play in the potential beneficial effects of music listening in couples?

### Research Questions and Hypotheses

By means of an ambulatory assessment study, it was investigated whether music listening in couples could have stress-reducing effects on both partners (Hypothesis 1) with partners experiencing less stress, if they themselves or their partner had listened to music. More specifically, it was hypothesized that in both partners music listening affected subjective stress, HPA axis activity, and ANS activity. It was tested in an exploratory manner, whether music preferences or similarity in the couples’ music preferences would moderate the beneficial effects of music listening (Hypothesis 2).

## Methods

This study was part of a larger project on the neuroendocrine mechanisms of couple interaction in which couples were randomly assigned to one of four conditions in a two by two design. The first independent variable was the daily administration of oxytocin or placebo, the second independent variable was the instruction to either engage in a positive couple interaction or no further interaction.

As the focus of the present analysis is on music and stress in everyday life, the current analyses are based on the placebo group only (n = 40 heterosexual couples). At the beginning of the study, half of the couples had received the instruction to engage in a 5–10 minute positive couple interaction (a couple appraisal task which included positive affirmations about the partner and the relationship). The couples in the positive interaction group were asked to practice this positive interaction at least twice during the study, while the other couples did not receive such an instruction. This intervention did not affect the results on music listening or the relationship between music listening and stress in the couples (p > 0.05).

### Participants

A total of n = 40 heterosexual couples (n = 80 individuals) were examined. The mean age was $\bar{{\rm{x}}}$ = 27.72 ± 5.30 years (men: $\bar{{\rm{x}}}$ = 28.71 ± 5.30, women: $\bar{{\rm{x}}}$ = 26.74 ± 5. 18). Among the rigid inclusion and exclusion criteria for the total sample were: fluent German, 21–45 years of age, being in a heterosexual exclusive relationship > one year and <15 years, sharing the same household, no children, BMI > 17 and <30, fewer than five cigarettes per week, no current drug consumption, no daily intake of alcohol exceeding 60 g alcohol/day, no intake of medication (except hormonal contraceptives), no acute or chronic somatic, neurological or psychiatric illness, and additionally for females: no pregnancy, no breast-feeding. Half of the female sample (N = 20) were taking hormonal contraceptives, half were naturally cycling. The mean relationship duration was $\bar{{\rm{x}}}$ = 3.72 ± 2.52 years. Couples reported living together on average since $\bar{{\rm{x}}}$ = 1.99 ± 1.67 years. Participants were recruited via advertisements in local newspapers, notices on local bulletin boards and online. All participants provided informed consent. Participation in the one-week clinical trial was voluntary and each couple received 500 CHF as compensation. The study was in line with the Declaration of Helsinki and approved by the Ethics Committee of the Canton of Zurich, Switzerland.

### Procedures

The study was designed as an ambulatory assessment study in which couples were examined for five consecutive days in their daily life. Initially, a telephone-based interview was conducted in order to screen for the eligibility criteria. In case of eligibility, couples were invited to the University Hospital Zurich, Switzerland, for an introductory session. During this introductory session, a pregnancy test as well as a multi-drug test were performed. To take into account possible influences of sex-hormones on stress, cortisol or alpha-amylase, all investigations were scheduled during the women’s early follicular phase (day 03–08 of the menstrual cycle), when estradiol and progesterone are low. Partners filled out questionnaires on relationship quality, stress, and music preference, individually. Afterwards, participants were familiarized with the handling of a pre-programmed iPod®touch (iDialogPad), on which they were required to complete six assessments on each day for the following five days. The first assessment had to be initiated by the participant directly after awakening. Subsequently, a timer activated the next assessments at 30 min, 150 min, 480 min and 720 min after awakening. The sixth assessment had to be triggered by the participants directly before going to bed. Additionally, participants were instructed to provide a saliva sample (Salicaps® IBL, Hamburg, Germany) at each assessment for the later analysis of salivary cortisol (sCort) and salivary alpha-amylase (sAA). They were instructed on how to collect and store the saliva samples. These assessments were scheduled to start the day after the introductory session.

### Measures

Relationship quality, music preferences, and stress were all assessed as (1) trait variables at baseline, and (2) as momentary variables during the ambulatory assessment procedures.

#### Baseline Questionnaires

Both partners were asked to fill in the (were asked to fill in the German version of the marital quality questionnaire (PFB; Partnerschaftsfragebogen^33^ on partnership quality. The PFB comprises 30 items covering the three scales ‘communication/togetherness’, ‘tenderness’, and ‘conflict behavior’.

Participants reported their music preference, using the “Music Preference Questionnaire” (MPQ)^34^. The MPQ (comprises) among others items on music preferences in terms of preferred music genres (10 items), preferred reasons for music listening (10 items), preferred situations in which music is listened to (4 items), current and past musical activities (2 items), as well as importance of music for one’s own life (1 item). With the exception of items on current and past musical activities, agreement with each item was scored using a 5-point Likert scale with higher scores represent higher agreement with the respective item. Current and past musical activities are assessed by dichotomous items (yes vs. no). The MPQ is characterized by high face validity. We compared the mean preference scores for each music genre in this study to the descriptive values of the MPQ based on a survey covering n = 1182 participants^35^. There were no significant differences in mean preference scores for each music genre (all p > 0.05). The MPQ was used in order to gain insights into the similarity of the couples’ music preferences. The similarity of music preferences was determined by calculating the mean deviance between the preference for each item group (e.g., items on preference for music genres).

Chronic stress was assessed using the short version of the “Trier Inventory for Chronic Stress” (TICS-S), which is derived from the first revision of the Trier Inventory for Chronic Stress (TICS)^36^. Ten scales (e.g., ‘work overload’, ‘social overload’) are covered by three items each, resulting in a total of 30 items with higher sum scores representing higher chronic stress.

#### Momentary Self Report Data

Participants provided six daily measures for five days. However, as the first assessment (which was triggered directly after awakening) did not contain items on music listening, this first assessment will be omitted from all subsequent analyses.

Music listening behavior was assessed five times daily via self-report using a single-item approach. During each assessment (+30, +150, +480, +720, directly before going to bed), participants were asked whether they had listened to music since the last assessment. This item could either be answered ‘yes’ or ‘no’.

At four assessments (+150, +480, +720, directly before going to bed), participants reported how stressed they felt at the moment on a five-point scale ranging from relaxed (1) to stressed (5). The momentary assessment of subjective stress using a single-item approach has previously been validated^37^.

An overview on the distribution of items on time of assessment can be found in Supplemental Materials (Appendix A).

#### Physiological Measures

Participants were asked to provide a saliva sample at each assessment for the analysis of sCort and sAA. Cortisol is the end-product of the hypothalamus-pituitary-adrenal (HPA) axis, with cortisol increases representing increases in HPA axis activity. The activity of sAA is associated with increased autonomic nervous system (ANS) – particularly sympathetic – activation. Both sCort and sAA can be reliably and validly assessed from saliva^3,38^. Using pre-labeled SaliCaps® (IBL, Hamburg, Germany), participants had to accumulate saliva for about one minute which was monitored by the electronic diary device by means of a countdown counting backwards. They were asked to let it passively drool into the SaliCaps®. In order to monitor compliance in this study, all SaliCaps had a specific code and study participants were asked to enter the number of the respective SaliCap into the electronic diary device. Thus, we had the exact time available when each assessment was done and controlled for this in subsequent analysis. As a further validation of compliance, we checked with the individual cortisol day curves, which are a valid indicator of when study participants have provided saliva samples during the course of the day (Table 1). Participants were asked to store the saliva samples in their fridge at home (at around 7 °C). Upon return to the laboratory after five days, the saliva samples were stored at −20 °C until analyses.

**Table 1:** Descriptive statistics on salivary cortisol and salivary alpha-amylase.

	T1	T2	T3	T4	T5	T6
	M ± SD	M ± SD	M ± SD	M ± SD	M ± SD	M ± SD
cortisol in nmol/l	11.50 ± 5.83	14.67 ± 7.43	8.47 ± 5.70	4.97 ± 3.71	3.35 ± 3.64	3.05 ± 4.76
alpha-amylase in U/ml	117.42 ± 120.35	46.08 ± 54.34	94.43 ± 100.25	119.82 ± 112.50	109.10 ± 104.02	84.53 ± 97.84

Annotations: T1: directly after awakening, T2: 30 minutes after awakening, T3: 2.5 hours after awakening, T4: 8 hours after awakening, T5: 12 hours after awakening, T6: directly before going to bed, M: mean, SD: standard deviation.

### Data Analysis

With this time series and nested structure of the data, hierarchical linear modeling (HLM^39^) was considered most suitable for data analysis^40^. As the data is characterized by dyadic interdependence, a multilevel model for dyadic diary data was used that treats the three levels of distinguishable dyadic diary data (days nested within persons nested within couples) as two levels of random variation^41^. This approach allows distinguishing within-dyad from between-dyad variations. Therefore, two kinds of transformations were done prior to analysis: First, overall centering of the predictor was performed (here: item on music listening behavior). Second, this predictor was separated into components reflecting within and between partner variation, separately for male and female partners (music listening (man), music listening (woman), music listening man in rows of woman and music listening of woman in rows of man). Furthermore, all analyses control for time of day due to the known diurnal variations in sCort^42^ and sAA^2^ and for body mass index (BMI)^43^. Biological data were checked for normality using the Kolmogorov—Smirnov test. Both sCort and sAA were log-transformed due to non-normality using the formula ln (x) + 10. In analyses including stress as outcome variable, stress measures from the previous assessment were included.

The overall model was tested by means of mixed model equations either using subjective stress, sCort or sAA as outcome measure (here the model concerning hypothesis 2 is presented). As all analyses controlled for the stress measure from the previous assessment using a lag-1 measure, the focus of the analyses is the phasic response of cortisol and alpha-amylase secretion induced by music listening during the time-window just before the assessment.

*LnCortisol*_*ij*_ = *γ00* + *γ*_01_*Diff_Gen_*j*_ + *γ*_10_*WsCort_p_*ij*_ + γ11*WBMI_j_*WsCort_P_*ij*_ + γ20*HsCort_P_*ij*_ + γ21*HBMI_j_*HsCort_p_*ij*_ + γ30*Female_*ij*_ + γ40*Male_*ij*_ + γ50*Htime_*ij*_ + γ60*Wtime_*ij*_ + γ70*Wmusic_*ij*_ + γ71*Diff_Gen_j_*Wmusic_*ij*_ + γ80*Hmusic_*ij*_ + γ81*Diff_Gen_j_*Hmusic_*ij*_ + γ90*HWmusic_*ij*_ + γ91*Diff_Gen_j_*HWmusic_*ij*_ + γ100*WHmusic_*ij*_ + γ101*Diff_Gen_j_*WHmusic_*ij*_ + *u0*_*j*_ + *r*_*ij*_.

#### Annotations

Diff_Gen: difference score in music preferences; WsCort_p: cortisol value of woman at previous assessment; WMBI: body-mass-index of woman; HsCort_p: cortisol value of man at previous assessment; Htime: time of assessment of man; Wtime: time of assessment of woman; Wmusic: music listening of woman (0 = no; 1 = yes); Hmusic: music listening of man (0 = no, 1 = yes); HWmusic: music listening of man in rows of woman ( = women, influenced through men’s values); WHmusic: music listening of woman in rows of man (=men, influenced through women’s values).

The dataset consisted of 40 (couples) * 2 (persons) * 5 (days) * 5 (assessments per day) = 2000 potential observations concerning analyses including music listening behavior, sCort, and sAA. However, as stress from the previous assessment was included using a lag −1 measure, all analyses are based on potential 1600 (sCort and sAA) or 1200 (subjective stress) observations. In case of more than 50% of missing values per person, respective participants have been excluded prior to data analysis.

P-values of ≤0.05 were considered significant. Unstandardized coefficients (UC) are presented.

## Results

### Baseline Questionnaires

According to data from the PFB on partnership quality, female partners rated relationship quality higher than their male partners ($\bar{{\rm{x}}}$_women_ = 74.05 ± 6.61, $\bar{{\rm{x}}}$_men_ = 71.58 ± 8.07, *t*(39) = 2.090, *p* = 0.043).

**Figure 1:**
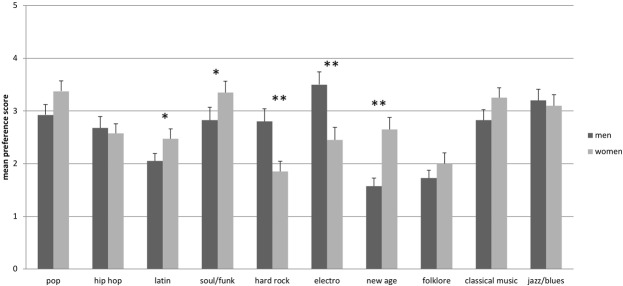
Mean preference scores for music genres, separately for men and women. Note: error bars represent standard error of the mean, *p ≤ 0.05, **p ≤ 0.001.

Data on music preference, as assessed by the MPQ, shows that music listening was reported as being important for both men and women (men: $\bar{{\rm{x}}}$ = 3.95 ± 0.93; women: $\bar{{\rm{x}}}$ = 4.10 ± 0.98). Preference for music genres differed between men and women (Fig. 1): Women showed significantly higher preferences for latin (p = 0.042), soul/funk (p = 0.043) and new age (p ≤ 0.001), whereas men showed higher preferences for hard rock (p ≤ 0.001) and electro (p ≤ 0.001). There were no differences in the habitual use of music listening for specific reasons between men and women (all p ≥ 0.087). Also, the importance of music did not differ between women and men (*t*(39) = −0.845, *p* = 0.403).

Interestingly, music preferences within couples were more similar to those of random heterosexual dyads generated from the data. For example, the preference scores for specific music genres differed between the female and the male partner ($\bar{{\rm{x}}}$ = |1.17| ± 0.40 scores), but this difference was significantly lower in couples in comparison to random dyads (*t*(39) = −3.471, *p* ≤ 0.001). Habitual use of music for specific reasons however differed within couples ($\bar{{\rm{x}}}$ = |1.30| ± 0.42), with no significant differences in comparison to random dyads (*t*(39) = −1.490, *p* = 0.144).

Concerning the experience of chronic stress as measured by the TICS-S, the sum score of each subscale varied between $\bar{{\rm{x}}}$ = 5.53 ± 2.05 (subscale ‘demands at work’) and $\bar{{\rm{x}}}$ = 8.99 ± 2.68 (subscale ‘performance pressure in social interactions’). Women scored higher on the subscales “work overload” (*p* ≤ 0.001), “social overload” (*p* = 0.020), “excessive demands at work” (*p* = 0.028), “lack of social recognition” (*p* = 0.049), and “worry propensity” (*p* ≤ 0.001).

#### Momentary Self-Report Data

Music listening was reported at 25.0% of all measures (women: 24.4% of time points, men: 25.6% of time points). The mean stress level experienced was $\bar{{\rm{x}}}$ = 2.23 ± 0.98 with women reporting higher stress than men (*t*(1576) = −4.733, *p* ≤ 0.001).

### Subjective Stress Measures

#### Associations Between Music Listening and Subjective Stress

First, it was tested whether partners perceived less stress, if they themselves had listened to music and/or if their partner had listened to music since the previous data entry. The unconditional model included subjective stress as outcome variable, subjective stress at previous assessment, and time since awakening at level-1 and control variables at level-2. The conditional model was specified by adding music listening (man), music listening (woman), music listening man in rows of woman (= women, influenced through men’s values), (music listening) of (man...), and music listening of woman in rows of man (= men, influenced through women’s values) as predictors. There was no effect of music listening or the partner’s music listening on own subjective stress, neither for women nor for men (Table 2).

**Table 2:** Hierarchical linear modeling predicting repeatedly assessed stress, salivary cortisol, and salivary alpha-amylase by music listening using restricted maximum likelihood.

Fixed effects	Changes in subjective stress		Changes in salivary cortisol		Changes in salivary alpha-amylase	
	UC	SE (df), *p*	UC	SE (df), *p*	UC	SE (df), *p*
Women intercept	−0.84	*0*.*85 (1104)*, *0*.*320*	*1*.*12*	0.73 (1752), *0*.*125*	0.58	0.92 (1752), *0*.*528*
Men intercept	−1.04	0.89 (1104), *0*.*241*	−0.98	0.97 (1752), *0*.*313*	0.90	1.08 (1752), *0*.*406*
Time since awakening (women, within-effect)	−0.00	0.00 (1104), ≤*0*.*001*	−0.00	0.00 (1752), ≤*0*.*001*	0.00	0.00 (1752), ≤*0*.*001*
Time since awakening (men, within-effect)	−0.00	0.00 (1104), ≤*0*.*001*	−0.00	0.00 (1752), ≤*0*.*001*	0.00	0.00 (1752), *0*.*128*
Stress^a^ at previous assessment (women, within)	0.29	0.03 (1104), ≤*0*.*001*	0.25	0.05 (1752), ≤*0*.*001*	0.15	0.05 (1752), ≤*0*.*001*
BMI^b^ (women, within)			0.00	0.00 (1752), *0*.*941*	0.01	0.00 (1752), ≤*0*.*001*
Stress1 at previous assessment (men, within)	0.29	0.04 (1104), ≤*0*.*001*	0.38	0.05 (1752), ≤*0*.*001*	0.31	0.05 (1752), ≤*0*.*001*
BMI2 (men, within)			0.00	0.00 (1752), *0*.*405*	0.00	0.00 (1752), *0*.*810*
Music episode (women, within) (0/1)^b^	−0.06	0.08 (1104), *0*.*451*	−0.13	0.06 (1752), *0*.*022*	−0.06	0.07 (1752), *0*.*451*
Music episode (men, within) (0/1)^c^	0.06	0.08 (1104), *0*.*446*	−0.04	0.06 (1752), *0*.*549*	0.23	0.07 (1752), *0*.*002*
Music episode (women, influenced through men’s values)	0.08	0.08 (1104), *0*.*293*	0.07	0.06 (1752), *0*.*259*	0.17	0.07 (1752), *0*.*022*
Music episode (men, influenced through women’s values)	0.01	0.08 (1104), *0*.*930*	−0.15	0.06 (1752), *0*.*010*	0.28	0.07 (1752), ≤*0*.*001*

Note: ^a^In model a: subjective stress, in model b: salivary cortisol, in model c: salivary alpha-amylase, ^b^BMI was only controlled for in analyses concerning salivary cortisol and salivary alpha-amylase, ^c^(0/1): 0 = no music listening, 1 = music listening, SE: standard error, df: degrees of freedom, UC Unstandardized Coefficient.

### Physiological Measures

#### Associations Between Music Listening and Physiological Markers of Stress

**Figure 2:**
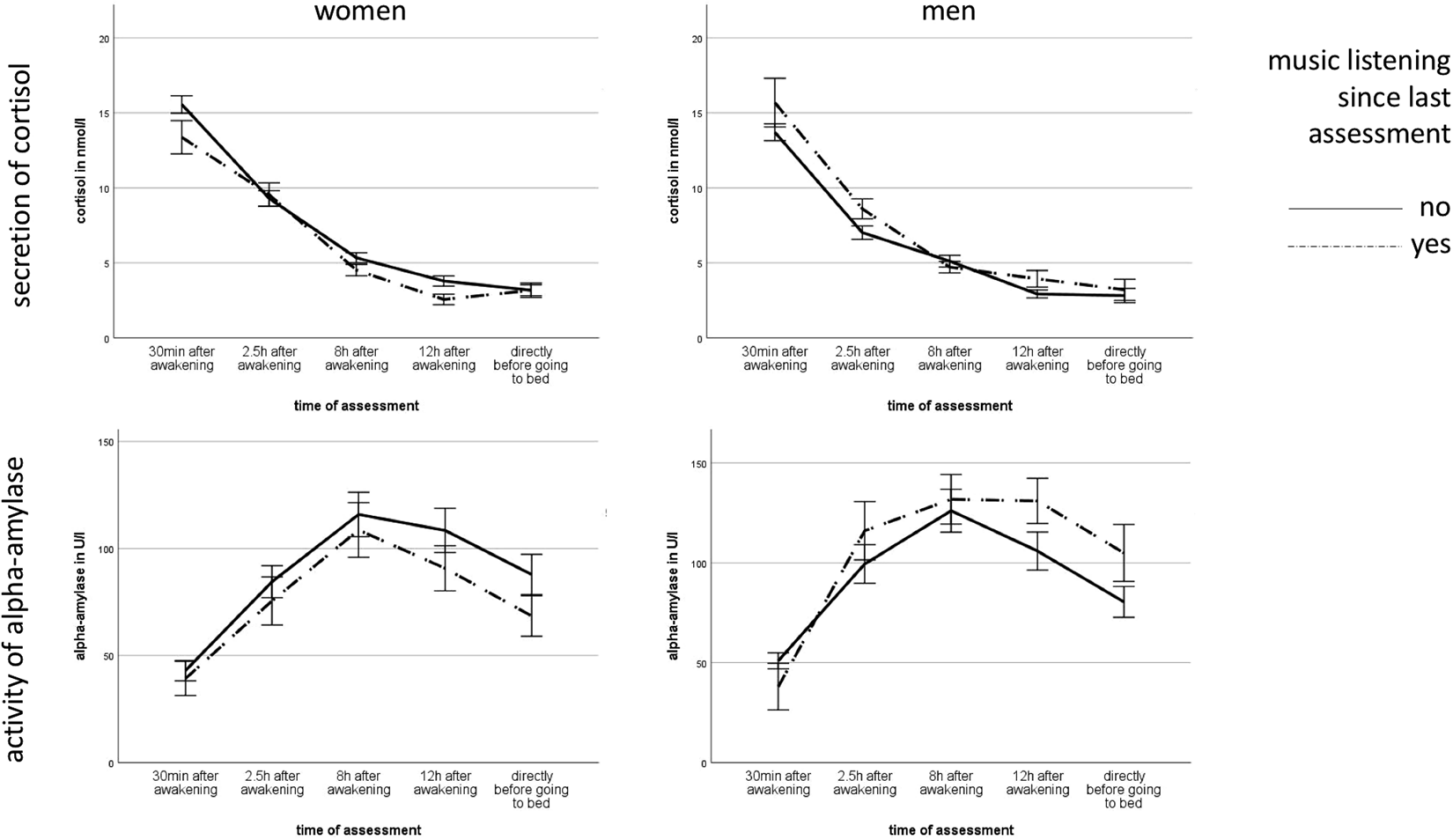
Mean secretion of cortisol and activity of alpha-amylase in relation to music listening in women and men. Annotations: error bars represent ±1 standard error of the mean.

Individually seen, women showed lower secretion of sCort, if they themselves had listened to music (*UC* = −0.13, *t*(1752) = −2.294, *p* = 0.022), whereas men showed higher sAA activity after music listening (*UC* = 0.23, *t*(1752) = 3.121, p = 0.002) (Fig. 2). Concerning dyadic effects, men showed lower secretion of sCort (*UC* = −0.15, *t*(1752) = −2.584, *p* = 0.010) and higher activity of sAA (*UC* = 0.28, *t*(1752) = 3.771, *p* ≤ 0.001), if their partner had listened to music. Furthermore, women showed higher sAA activity, when their partner had listened to music (*UC* = 0.17, *t*(1752) = 2.294, *p* = 0.022). All results can be found in Table 2.

As the music preference for latin, soul, new age, hard rock, and electro differed between women and men, analyses controlled for the similarity of music preference on level-2. Subjective stress after listening to music was not influenced by the similarity of music preferences (women: *UC* = −0.01, *t*(1100) = −0.628, *p* = 0.530; men: *UC* = −0.02, *t*(1100) = −0.887, *p* = 0.375), however the above listed cortisol responses were. Men showed lower sCort secretion after their female partner listened to music, particularly if the music preferences were similar (*UC* = 0.06, *t*(1748) = 3.481, *p* ≤ 0.001). Concerning the activity of sAA, no associations were found (*UC* ≤ 0.04, *t*(1786) ≤ 1.627, *p* ≥ 0.104).

## Discussion

### Summary of Results

The present study found associations between music listening and psychobiological stress in couples with evidence for dyadic transfer of music-induced physiological changes from one individual to another, however in a sex-specific manner. More precisely, both women and men showed lower secretion of sCort, if the female partner had listened to music. In men, higher sAA activity was found when they themselves had listened to music or their female partner had. Women showed higher sAA activity when the male partner had listened to music however no within-association of music and sAA was found in women. Concerning co-variation in the secretion of sCort, these physiological effects were more pronounced when both partners shared similar (instead of the same) music preferences and, indeed, couples showed higher overall convergence in music preferences, as compared to scrambled dyads. In this present sample, subjective stress levels did not vary depending on music listening in dyads.

### Sex Differences in the Stress-Reducing Effect of Music Listening

Sex differences in psychophysiological responses to music listening have been reported elsewhere^44,45^. Gupta and Gupta^44^ describe that music listening leads to a reduction of blood pressure in women, whereas no such effect was found for men. Furthermore, there are sex differences in music preferences and reasons for music listening^46^. North^46^ reports that women are more likely to use music among others for relaxation whereas men choose music for reasons such as being creative. Our data are in line with such sex differences with reduced sCort levels in women after they themselves had listened to music and no such effects in men. Furthermore, our data points towards the idea that women seem to use music as a tool for stress reduction, whereas men listen to music as a means to stimulate themselves. Thus, men showed higher sympathetic arousal (sAA-levels) to music in our study leading to the assumption that music has rather arousal-provoking effects in men and stress-reducing effects in women in our study potentially related to sex differences in music selection and music preferences.

These results are of particular interest in the context of sex-specific modulation of stress in everyday life: Both men and women use different strategies to either up-regulate or down-regulate psychophysiological stress levels^47^ in order to reach an optimum in activation^48^. Although in our study, music listening was not significantly associated with subjective stress levels, the descriptive results point towards stress-reduction in women and increased stress in men (with a negative association of music and stress in women and a positive association in men). This fits with previous results where positive couple interaction was associated with reduced sAA levels in women and increased levels in men^49^. Based on this, it might be followed that the reduction of stress is not in the focus of both sexes per se, but that they seek to reach an individual set point of arousal. Women and men seem to use music for different reasons: Women as a stress-reducing activity, men for activation and stimulation.

In experimental studies sex differences in physiological reactions to music have been reported^50^ although the exact mechanisms underlying these differences remain to be elucidated. One line of explanation might link these differences to stable sex differences in musical preferences. North *et al*.^51,52^ complement this view, as they additionally elucidate situational variance in music preference based on arousal functions supporting our notion that in daily life physiological reaction to music might depend on the individual effort to achieve an optimum in activation with men and women potentially differing in their individual set point of arousal. This finding stands in accordance with findings from a diary study from Schäfer *et al*.^53^ who found that the strength of music preference is closely associated with the functions music fulfills (e.g. arousal regulation).

Thus, there seem to be relevant interactions between music preference, music functions, and sex differences in daily life concerning psychophysiological effects of music listening. Future studies need to further disentangle these interactions in order to understand mechanisms underlying the stress-reducing effects of music listening depending on factors associated with the person, the situation, and the music. Especially in daily life (in contrast to experimental studies) this knowledge is of utmost importance to tailor interventions for stress-reduction purposes in daily life with high ecological validity.

### Transmission of the Effects of Music Listening in Dyads

Both partners showed reduced secretion of sCort when the female partner had listened to music and both partners showed higher activity of sAA when the male partner had listened to music. Thus, the relaxing effects of music listening in women seem to translate to their partner if they themselves listen to music, whereas music has activating effects in men that translate to their partner. This suggests synchronization in stress-sensitive systems between partners. When the female partner experienced a stress-altering effect of music listening on a psychobiological level, this effect translated to the partner and vice versa. This finding is interesting, especially against the background of physiological linkage in dyads.

Physiological linkage refers to patterns of co-variation in physiological states among different people^54^. Such co-variation can either occur in unison (in-phase changes) or in changes in opposite direction (anti-phase changes)^55^. To date it is subject to debate whether overall physiological linkage has positive or negative effects in couples^54^. Our results suggest that on a psychobiological level, sex-specific analyses might provide more specific information. If men and women co-regulate with their partner coming from different levels of arousal, this might not necessarily be beneficial for both of them. In this context, it is interesting that in our data the linkage in HPA axis was further enhanced when similar music preferences were reported. This suggests that joint music preferences have an important influence on psychobiology in everyday life, as these might pre-determine the couple’s choice of music towards either relaxation or activation. Joint music preferences might be indicative of similar arousal preferences in daily life facilitating homeostatic interpersonal regulation through physiological linkage. Future experimental research might systematically ask couples to either choose relaxing or activating music, and then investigate psychobiological co-regulation processes. Complementing these findings in daily life, designs are necessary that allow tracking the exact music participants are listening to and to analyze the characteristics of the music, the person, and the situation^56,57^.

### Discrepancies between subjective and physiological effects of music listening

It is of note, that in accordance to previous studies on biopsychological mechanisms underlying the effects of music listening, we found discrepancies between subjective reports on stress and sCort secretion and sAA activity^8,9^. Whereas we did not find an effect of music listening on subjective stress, we found a down-regulation of HPA axis activity (as mirrored by lower sCort secretion) and up-regulation of ANS activity (as mirrored by higher sAA activity). To reconcile these findings, we argue that the different temporal dynamics of subjective stress, HPA axis and ANS activity relative to a stressor go along with different influences of music listening on stress-sensitive systems in the body. Whereas characteristics of the music are closely associated to the activity of the ANS that are processed at an early stage of musical processing, the HPA axis activity is modulated in a time-lagged manner going along with the later emotional processing of music in the brain^58^. Thus, as we concomitantly measure HPA axis and ANS activity, these relaxing and activating effects of music listening may reflect different stages of musical processing in addition to the effect of sex-specific music preferences and music selection.

### Limitations

Although this is the first study to examine the effects of music listening on stress in healthy couples encompassing both subjective and physiological markers of stress, certain limitations have to be critically acknowledged: First of all, it was only assessed whether music listening had occurred since the last assessment. Consequently, it can only be analyzed indirectly via data on the presence of the partner whether partners had listened to music together. It might be an important endeavor for future studies to investigate the effects of joint music listening on stress. Further, we are only able to investigate the effects of music listening since the last assessment and its effects on stress levels at the subsequent assessment without controlling for whether music listening occurred at assessments beforehand and without investigating how long the effects of music listening persisted as captured by subsequent stress levels. In order to evaluate the temporal dynamics underlying the stress-reducing effects in daily life, research designs are necessary that directly assess the time point of music listening and assess repeatedly over time biopsychological stress measures in relation to the time of music listening. Furthermore, we did not assess music genres participants were listening to during data collection. It would be intriguing to study in future studies whether differences in music genres account for the sex-specific differences in physiological effects of music listening. Also, as previous studies found the importance of context factors (e.g., reasons for music listening)^8^, future studies should additionally examine whether the context of music listening with regard to reasons for music listening, activities, places and/or motivation for music listening modulates the effects of music listening on psychobiological stress in couples via experience sampling methods that directly assess which music was listened to and link the characteristics of the music listening to context factors and personal preferences^59,60^. As we found effects to be varying depending on similarity of music preferences, it has to be critically discussed that the German measure we used for assessing music preference is characterized by high face-validity. Therefore, in future studies the findings on music preferences should be corroborated using validated measures for assessing music preferences (e.g., scale provided by Litle and Zuckerman^61^; STOMP as provided by Rentfrow and Gosling^62^).

## Conclusion

Music listening in daily life affects psychobiological stress experiences in couples. However, gender differences in these effects seem to apply. Whereas women showed lower HPA axis activation (as indicated by sCort secretion) after listening to music, males showed higher autonomic activity (indicated by sAA) after listening to music. These psychobiological patterns transferred to the partner with lower sCort secretion in men, if their female partner had listened to music and higher sAA activity in women, if their male partner had listened to music. The effect was particularly strong, when partners reported similar music preferences. Therefore, despite gender differences in the use of music, music listening seems to co-regulate the experience of stress in dyads. Particularly this latter finding has strong implications for the research on co-regulation in general: rather than the mere positive or negative interpretation of co-regulated systems, the beneficial effects of dyadic attunement might depend on the individual (and sex-specific) motives to either up- or down regulate psychobiological arousal. This understanding can affect music therapy and everyday life music listening alike.

## Supplementary information

Appendix A. Overview on distribution of items relative to time of assessment

## Data Availability

The datasets generated during and/or analyzed during the current study are available from the corresponding authors on reasonable request.
