# Publisher Correction: Sex-specific Effects of Music Listening on Couples’ Stress in Everyday Life

**DOI:** 10.1038/s41598-020-58620-4

**Published:** 2020-01-28

**Authors:** A. Wuttke-Linnemann, U. M. Nater, U. Ehlert, B. Ditzen

**Affiliations:** 1 University Medical Center Mainz, Mainz, Germany; 2 University of Vienna, Vienna, Austria; 3 University of Zurich, Zurich, Switzerland; 4 University Hospital, Heidelberg University, Heidelberg, Germany; 5 Center for Mental Health in Old Age, Landeskrankenhaus (AöR), Mainz, Germany

Correction to: *Scientific Reports* 10.1038/s41598-019-40056-0, published online 19 March 2019

This Article contains typographical errors.

In the introduction,

“Dyadic data analysis suggests that in couples each partner’s (that in couples each partner’s) evaluations of marital quality (are also modulated by the) stress (of their partner) are also modulated by the affect of their partner, a phenomenon referred to as stress crossover^16^.”

should read:

“Dyadic data analysis suggests that in couples each partner’s evaluations of marital quality are also modulated by the stress of their partner, a phenomenon referred to as stress crossover^16^.”

In the methods under subheading ‘Baseline Questionnaires’,

“Both partners were asked to fill in the (were asked to fill in the German version of the marital quality questionnaire (PFB; Partnerschaftsfragebogen^33^ on partnership quality. The PFB comprises 30 items covering the three scales ‘communication/togetherness’, ‘tenderness’, and ‘conflict behavior’. Participants reported their music preference, using the “Music Preference Questionnaire” (MPQ)^34^. The MPQ (comprises) among others items on music preferences in terms of preferred music genres (10 items), preferred reasons for music listening (10 items), preferred situations in which music is listened to (4 items), current and past musical activities (2 items), as well as importance of music for one’s own life (1 item).”

should read:

“Both partners were asked to fill in the German version of the marital quality questionnaire (PFB; Partnerschaftsfragebogen^33^). The PFB comprises 30 items covering the three scales ‘communication/togetherness’, ‘tenderness’, and ‘conflict behavior’. Participants reported their music preference, using the “Music Preference Questionnaire” (MPQ) ^34^. The MPQ comprises among others items on music preferences in terms of preferred music genres (10 items), preferred reasons for music listening (10 items), preferred situations in which music is listened to (4 items), current and past musical activities (2 items), as well as importance of music for one’s own life (1 item).”

Additionally, in the methods under subheading ‘Annotations’,

“Diff_Gen: difference score in music preferences; WsCort_p: cortisol value of woman at previous assessment; WMBI: body-mass-index of woman; HsCort_p: cortisol value of man at previous assessment; Htime: time of assessment of man; Wtime: time of assessment of woman; Wmusic: music listening of woman (0 = no; 1 = yes); Hmusic: music listening of man (0 = no, 1 = yes); HWmusic: music listening of man in rows of woman ( = women, influenced through men’s values); WHmusic: music listening of woman in rows of man (=men, influenced through women’s values).”

should read:

“Diff_Gen: difference score in music preferences; WsCort_p: cortisol value of woman at previous assessment; WBMI: body-mass-index of woman; HBMI: body-mass-index of man; HsCort_p: cortisol value of man at previous assessment; Htime: time of assessment of man; Wtime: time of assessment of woman; Wmusic: music listening of woman (0 = no; 1 = yes); Hmusic: music listening of man (0 = no, 1 = yes); HWmusic: music listening of man in rows of woman ( = women, influenced through men’s values); WHmusic: music listening of woman in rows of man (=men, influenced through women’s values).”

In the Results under subheading ‘Associations Between Music Listening and Subjective Stress’,

“The conditional model was specified by adding music listening (man), music listening (woman), music listening man in rows of woman (= women, influenced through men’s values), (music listening) of (man...), and music listening of woman in rows of man (= men, influenced through women’s values) as predictors.”

should read:

“The conditional model was specified by adding music listening (man), music listening (woman), music listening of man in rows of woman (= women, influenced through men’s values), and music listening of woman in rows of man (= men, influenced through women’s values) as predictors.”

Finally, in the Discussion under subheading ‘Summary of Results’,

“Concerning co-variation in the secretion of sCort, these physiological effects were more pronounced when both partners shared similar (instead of the same) music preferences and, indeed, couples showed higher overall convergence in music preferences, as compared to scrambled dyads.”

should read:

“Concerning co-variation in the secretion of sCort, these physiological effects were more pronounced when both partners shared similar music preferences and, indeed, couples showed higher overall convergence in music preferences, as compared to scrambled dyads.”

