# Supplementary material for: Sex-specific Effects of Music Listening on Couples’ Stress in Everyday Life
Source: Sci Rep. 2019 Mar 19;9:4880. doi: 10.1038/s41598-019-40056-0 (PMC6424968; doi:10.1038/s41598-019-40056-0)
Supplement: Supplementary file 1 — Appendix A. Overview on distribution of items relative to time of assessment [file 41598_2019_40056_MOESM1_ESM.pdf]

Supplemental Material

Sex-specific Effects of Music Listening on Couples' Stress in Everyday Life

Wuttke-Linnemann, A.[1], Nater, U. M.[2], Ehlert, U.[3] & Ditzen, B.[3,4]
[1] Mainz University Medical Center, Germany
[2] University of Vienna, Austria
[3] University of Zurich, Switzerland
[4] University Hospital, Heidelberg University, Germany

Corresponding author:
Beate Ditzen, PhD
Heidelberg University Hospital
Institute of Medical Psychology
Bergheimer Straße 20
69115 Heidelberg
beate.ditzen@med.uni-heidelberg.de
Phone: +49-6221-56-8150

and

Alexandra Wuttke-Linnemann, PhD
Mainz University Medical Center
Clinic for Psychiatry and Psychotherapy
Untere Zahlbacher Straße 8
55131 Mainz
alexandra.linnemann@unimedizin-mainz.de
phone: +49-6131-17-2488

Appendix A. Overview on distribution of items relative to time of assessment

**Time of assessment (relative to wake-up)**	**Music listening behavior[1]**	**Subjective stress[2]**	**cortisol[3] and sAA[4]**
Wake-up[5]			X
+30 minutes	X		X
+150 minutes	X	X	X
+480 minutes	X	X	X
+720 minutes	X	X	X
Directly before going to bed[6]	X	X	X

Annotations: *[1] music listening behavior was assessed using the item: 'Have you listened to music since the last assessment?' [2] subjective stress was assessed using the item: 'At this moment, I feel stressed' [3] salivary cortisol [4] salivary alpha-amylase [5] the first assessment had to be triggered directly after awakening [6] the sixth assessment had to be triggered directly before going to bed*
